# Beyond empirically supported treatments: a new contextualized evidence framework for evidence based psychology

**DOI:** 10.3389/fpsyt.2026.1819583

**Published:** 2026-05-28

**Authors:** Tamara Melnik, Ana Carolina Pereira Nunes Pinto

**Affiliations:** 1 Federal University of Sao Paulo, Sao Paulo, Brazil; 2 Institut de Recerca Sant Pau (IR Sant Pau), Barcelona, Spain; 3 Centro Cochrane Iberoamericano, Barcelona, Spain

**Keywords:** contextual factors, Empirically Supported Treatments (EST), evidence-based practice (EBP), health equity, implementation science, psychotherapy

## Abstract

Over the past three decades, criteria for Empirically Supported Treatments (ESTs) have strengthened methodological rigor in psychotherapy research by prioritizing randomized controlled trials and systematic evidence synthesis. However, prevailing frameworks remain largely centered on efficacy under controlled conditions, offering limited operational guidance for integrating contextual factors that critically shape real-world effectiveness. We propose the Contextualized Empirically Supported Treatment Framework (C-EST), a normative and operational model that integrates certainty of evidence with structured appraisal of contextual domains essential for clinical decision-making, guideline development, and policy. Building on the Tolin criteria, GRADE, GRADE-CERQual, and the GRADE Evidence-to-Decision approach, C-EST embeds assessment of empirical certainty within five interconnected domains (1): empirical evidence (2), critical appraisal of the body of evidence, (3) functional and cultural impact, (4) contextual factors, including values, acceptability, feasibility, and equity, and (5) transparency and living evidence. Rather than relying on binary classifications (“supported” vs. “unsupported”), the framework enables nuanced judgments such as “supported with contextual concerns,” explicitly documenting boundaries of applicability. Using Cognitive Behavioral Therapy for depression and psychological interventions for postpartum depression as illustrative cases, we demonstrate how contextual appraisal refines, rather than weakens, established evidence classifications. By aligning internal validity with external, cultural, and equity relevance, C-EST transforms evidence evaluation from a static designation into a dynamic decision-support tool. Integrating contextual evidence is not an ethical add-on but a methodological imperative to ensure that psychological treatments are not only efficacious, but applicable, equitable, and responsive to diverse real-world mental health needs.

## From empirical rigor to contextual relevance

Over the past three decades, the field of psychological treatment evaluation has undergone substantial methodological refinement. The criteria for Empirically Supported Treatments (ESTs), first published by the APA Division 12 Task Force in 1993 and later revised by Tolin et al. (2015) (1), established rigorous standards for identifying interventions supported by empirical evidence. These criteria played a foundational role in advancing evidence-based practice in mental health by emphasizing randomized controlled trials, replicability, and transparency.

As the evidence-based movement matured, the scope of evidence appraisal expanded beyond individual trials. The 2015 revision of the EST model explicitly acknowledged the importance of systematic review methods and encouraged the use of the GRADE approach to assess the certainty of evidence. In parallel, broader evidence synthesis frameworks, such as GRADE and the Joanna Briggs Institute (JBI) model, introduced structured methods for incorporating domains traditionally treated as peripheral in psychotherapy research, including patients’ values and preferences, acceptability, feasibility, and equity considerations. Together, these developments signaled a shift toward more comprehensive and transparent approaches to evaluating interventions (2, 3).

Despite these advances, psychological treatment evaluation continues to face challenges that complicate the translation of empirical findings into real-world practice. Psychotherapy research is characterized by theoretical pluralism, interpersonal processes, and substantial variability across therapists, patients, and settings. Blinding is often infeasible, standardization is limited, and outcomes prioritized in trials frequently emphasize symptom reduction over functioning, well-being, or outcomes valued by patients. As a result, interventions supported by high-quality efficacy evidence may show attenuated or inconsistent effects when implemented in routine care, particularly across diverse cultural, social, and economic contexts.

Although contextual and cultural factors are increasingly recognized as relevant to evidence-based mental health care, no framework currently offers a structured and operational method for integrating these dimensions into the evaluation of psychological treatments. Despite decades of methodological refinement, existing approaches remain largely anchored in efficacy under controlled conditions, creating a persistent gap between what is considered “empirically supported” and what can be effectively delivered, accepted, and sustained in real-world mental health care. Unlike implementation frameworks such as PARIHS, which focus on how to implement interventions, C-EST addresses how evidence should be appraised and classified before implementation decisions are made (4).

Ignoring contextual evidence is not a neutral methodological choice (5, 6). Empirical studies have shown that interventions with strong efficacy evidence often fail to achieve similar outcomes in routine care settings, particularly among underserved populations (7). Ignoring this systematically biases judgments of effectiveness toward populations, settings, and outcomes that are easiest to study, while marginalizing those most affected by mental health inequities. As a result, treatments classified as empirically supported under controlled conditions may remain inaccessible, unacceptable, or ineffective for populations facing structural barriers, thereby perpetuating unmet mental health needs. In this sense, unmet needs are not merely service delivery failures, but reflect methodological blind spots embedded within traditional evidence appraisal models.

This paper proposes the Contextualized Empirically Supported Treatment Framework (C-EST), a normative and operational model that integrates certainty of evidence with contextual domains essential for clinical decision-making, guideline development, and policy. By explicitly linking empirical rigor with applicability, equity, and implementation relevance, the C-EST framework reframes what it means for a psychological treatment to be considered “supported by evidence.” It operationalizes contextualized evidence appraisal in mental health by integrating internal validity with external, cultural, and ethical relevance.

## Bridging the gap: the integrated Tolin+ framework

The revision of the Empirically Supported Treatment criteria by Tolin et al. (2015) (1) marked an important methodological advance by formally incorporating systematic review standards and encouraging the use of the GRADE approach to assess the certainty of evidence. More recently, the forthcoming manual from the Society of Clinical Psychology (Division 12) further refines these criteria by providing detailed guidance on literature search strategies, documentation of treatment fidelity, transparency procedures, and risk-of-bias assessment (8). Together, these developments strengthen the internal validity and methodological rigor of psychological treatment evaluation.

However, even in their updated form, the Tolin criteria remain largely centered on efficacy evidence derived from controlled trials. While methodological rigor is essential, this trial-centric orientation offers limited operational guidance on how to incorporate contextual factors that critically shape real-world effectiveness, such as cultural relevance, acceptability, feasibility, and equity. As a result, treatments may be classified as empirically supported without adequate consideration of whether they can be meaningfully delivered, accessed, or sustained across diverse settings and populations.

The Contextualized Empirically Supported Treatment Framework (C-EST) is designed to address this gap by explicitly integrating the assessment of empirical certainty with structured appraisal of contextual domains. Rather than treating methodological rigor and contextual relevance as separate or competing considerations, the framework conceptualizes them as complementary dimensions of evidence validity. Certainty of evidence establishes whether an intervention produces meaningful effects, while contextual appraisal determines whom, under what conditions, and with what implications for equity those effects are likely to occur.

To operationalize this integration, the C-EST framework embeds the assessment of certainty of evidence, using GRADE for quantitative evidence and GRADE-CERQual for qualitative evidence, within a broader evaluative process informed by the GRADE Evidence-to-Decision (EtD) criteria and the Joanna Briggs Institute (JBI) model of evidence-based healthcare (2, 3, 9). This structure enables transparent and reproducible judgments that extend beyond efficacy to include patient-valued outcomes, implementation constraints, and equity considerations. Feasibility considerations may arise at multiple levels, including individual (e.g., time, cost), organizational (e.g., workforce capacity, service delivery), and system-level factors (e.g., scalability and resource allocation), consistent with established implementation science frameworks such as the Consolidated Framework for Implementation Research (CFIR) and RE-AIM (10, 11).

Empirical evidence from systematic reviews of values and preferences demonstrates that individuals consistently assign different levels of importance to specific outcomes (e.g., mortality reduction, false negative results, complications, and emotional burden), and are willing to make explicit trade-offs between benefits and harms. These preferences can be measured using a range of methods, including health state utility values (e.g., EQ-5D, time trade-off, standard gamble), discrete choice experiments, and qualitative approaches. This variability and structure in preferences reinforces the need for systematic and transparent incorporation of values and preferences into evidence appraisal frameworks.

While GRADE informs certainty of evidence and decision-making, it does not provide a treatment classification system specific to psychotherapy. The Contextualized Empirically Supported Treatment Framework (C-EST) addresses this gap by translating certainty assessments and contextual evidence into explicit psychotherapy-relevant classifications that reflect both empirical support and real-world applicability. It mandates transparent documentation of contextual boundaries and complements rather than replaces GRADE, by integrating quantitative and qualitative evidence into classification decisions, functioning as a bridge between evidence synthesis and clinical decision-making in mental health.

By consolidating these approaches into a single evaluative pathway, the C-EST framework moves beyond binary classifications of “supported” versus “unsupported” treatments. Instead, it allows interventions to be classified according to both the certainty of their effects and the contextual conditions under which those effects are applicable. This shift transforms evidence evaluation from a static designation into a dynamic decision-support tool for clinicians, guideline developers, and policymakers.

Equity has also been articulated as a core principle of the guideline enterprise, reinforcing the need for systematic consideration of how recommendations affect different populations (12, 13). However, such principles are rarely translated into explicit, operational criteria within evidence appraisal frameworks, highlighting a persistent gap between normative commitments and methodological practice.

The C-EST framework requires explicit judgment across five interconnected domains that jointly inform the classification of psychological treatments. These domains are not optional considerations or *post hoc* modifiers. Rather, they constitute essential components of evidence appraisal that determine both the certainty and the applicability of intervention effects.

Empirical evidence - Quantitative assessment of efficacy and precision, including magnitude and consistency of effects.

Critical appraisal of the body of evidence - Evaluation of bias, fidelity, and transparency, using validated RoB tools specific to the study design and following GRADE and JBI standards.

Functional and cultural impact - Assessment of outcomes beyond symptom reduction, including functioning, well-being, and cultural responsiveness.

Contextual factors, evidence and judgments (GRADE EtD and JBI Domains) - Appraisal of contextual factors, preferably considering the available evidence from either quantitative or qualitative studies on interest-holders’ values and preferences, acceptability, feasibility, and equity-related factors.

Acceptability, feasibility, and equity should be assessed using evidence from qualitative syntheses, implementation and observational studies, and, when available, mixed-methods research, capturing multilevel influences ranging from individual experiences to organizational and system-level constraints. Equity considerations should account for structured factors associated with health inequities, such as those captured by the PROGRESS-Plus framework (14) (e.g., place of residence, socioeconomic status, gender, and other social determinants of health).

5. Transparency, reproducibility, and living evidence - Ensure accountability and continuous improvement in evidence evaluation.

Together, these five domains form an integrated evaluative pathway rather than a linear or hierarchical checklist. Judgments within each domain are explicitly documented and considered jointly to inform the final classification of a psychological treatment. High certainty of evidence for efficacy does not, on its own, warrant an unqualified classification if substantial limitations are identified in contextual domains such as acceptability, feasibility, or equity. Such variability across populations, settings, and contexts reinforces the need for systematic contextual appraisal within evidence evaluation, rather than relying solely on implementation-stage considerations. For instance, variability in contextual factors across populations may lead to preference-sensitive decisions, supporting conditional recommendations even in the presence of high-certainty evidence of effect.

Within the C-EST framework, the classification “supported with contextual concerns” is assigned when moderate or high certainty of evidence of benefit is accompanied by at least one substantively limiting contextual domain (e.g., acceptability, feasibility, equity, or indirectness). To enhance operational clarity, contextual limitations should be explicitly judged using predefined signaling questions and categorized as “no or minor concerns”, “moderate concerns”, or “serious concerns”, following a structure analogous to GRADE Evidence-to-Decision criteria. Judgments are based on the direction, magnitude, and consistency of concerns identified across available evidence, rather than fixed quantitative thresholds. These decision rules are specified *a priori* to ensure reproducibility and to avoid *post hoc* or subjective classification. As with other evidence-to-decision frameworks such as GRADE, these judgments are informed by available evidence and guided by predefined signaling questions, and are expected to evolve through use, peer review, and scholarly dialogue, contributing to the progressive refinement and standardization of the framework. This approach ensures transparency, reproducibility, and consistency with established evidence-to-decision frameworks. Classification as “supported with contextual concerns” is warranted when at least one contextual domain is judged as presenting moderate or serious concerns.

**Figure 1:**
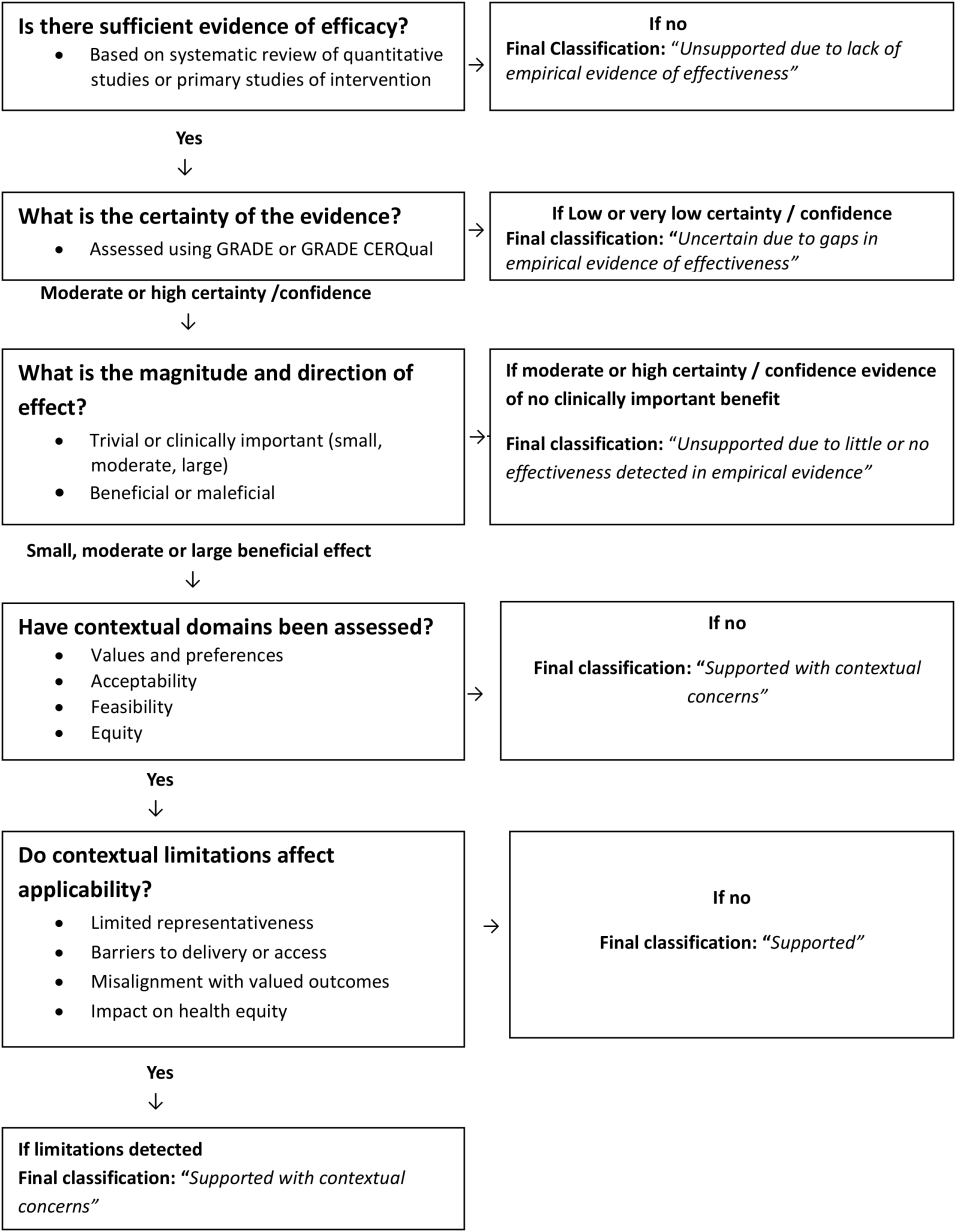
Flowchart of the contextualized EST framework.

Conversely, contextual relevance cannot compensate for very low certainty or absence of evidence of effectiveness. The C-EST framework thus requires transparent balancing of empirical certainty and contextual applicability to support informed, implementation-relevant decisions. Final outputs of the C-EST framework should include (1): classification label; (2) summary of certainty of evidence; (3) explicit documentation of contextual judgments across domains; (4) implications for implementation. **Figure 1** provides a simplified representation of the decision pathway. Detailed operational guidance, including signaling questions and judgment criteria, is provided in **Supplementary Table 1**. Judgments within these domains are based on the synthesis of available quantitative and qualitative evidence, using signaling questions adapted from GRADE Evidence-to-Decision frameworks, and require explicit justification to ensure transparency and reproducibility.

## Illustrating the shift: when context changes the evidence

Traditional EST models have often privileged internal validity over external or cultural relevance. This has led to a recurring paradox: interventions that are “well established” in controlled research sometimes show limited effectiveness in real-world practice, among diverse populations.

Consider the example of Cognitive Behavioral Therapy (CBT) for depression. CBT has one of the most extensive evidence bases in psychotherapy and is widely regarded as a well-established and empirically supported treatment for depression (15, 16). However, most CBT trials have been conducted with relatively homogeneous samples, typically middle-class, White, English-speaking participants with few comorbidities. When CBT is implemented in community settings or among ethnically diverse populations, its effectiveness often decreases (17). Socioeconomic stressors, cultural interpretations of distress, and differing help-seeking behaviors can all moderate treatment outcomes. Standard CBT protocols may inadvertently fail to address these contextual variables, reducing both engagement and effectiveness.

Applying the Integrated Framework to this case yields a richer and more nuanced appraisal. Under the traditional EST framework, CBT would likely remain classified as “well established.” However, when analyzed through GRADE’s EtD criteria and JBI’s contextual appraisal (18), reviewers may identify several limitations.

To enhance transparency, the appraisal follows an explicit evidence-to-decision pathway:

Step 1: Identification of evidence base.

Systematic reviews and meta-analyses of randomized controlled trials (15, 16) were used as the primary evidence base, representing the most comprehensive syntheses of CBT for depression.

Step 2: Certainty of evidence assessment (GRADE).

Moderate to high certainty of evidence supports reductions in depressive symptoms based on multiple randomized controlled trials and meta-analyses (15, 16). Effect sizes are generally small to moderate, with heterogeneity across delivery formats and populations. Some analyses report reduced effects in routine care and more diverse populations, suggesting lower effectiveness outside controlled settings.

Step 3: Assessment of contextual domains (GRADE EtD).

Judgments across contextual domains were informed by evidence from quantitative and qualitative studies, including cultural adaptation research, implementation studies, and observational data.

Values and preferences: partial misalignment with patient-valued outcomes. Evidence suggests that, in addition to symptom reduction, patients often prioritize functional improvement and meaningful life outcomes, while also expressing concerns related to treatment burden and accessibility. This indicates variability in outcome importance and supports at least moderate concerns in this domain.

Acceptability: variability across cultural groups. Cultural adaptation studies and reviews (17) indicate differences in engagement, satisfaction, and perceived relevance of CBT across ethnically diverse populations. Acceptability can be understood as a multifaceted construct encompassing factors such as perceived burden, ethicality, and intervention coherence (19), supporting moderate concerns regarding acceptability.

Feasibility: limited access in low-resource settings. Observational and implementation studies report barriers related to access to trained therapists, time constraints, and resource limitations in routine care, supporting moderate to serious feasibility concerns. Variability in therapist adherence and competence, as well as therapist drift from treatment protocols, has been documented and may contribute to heterogeneity in treatment outcomes (20).

Equity: underrepresentation of disadvantaged populations. The predominance of trials conducted in relatively homogeneous populations supports concerns regarding generalizability and potential inequities in access and outcomes (5, 17).

Together, this body of evidence justifies classifying these domains as presenting moderate to serious concerns.

Step 4: Final classification.

Supported with contextual concerns.

The classification “supported with contextual concerns” was assigned because, despite moderate to high certainty of evidence of benefit, at least one contextual domain (acceptability, feasibility, and equity) presented moderate to serious concerns based on available evidence, in accordance with the predefined decision rules of the C-EST framework.

This stepwise structure makes the classification process transparent, reproducible, and aligned with established evidence-to-decision frameworks, while allowing flexibility for context-sensitive interpretation across diverse populations and settings.

- Indirectness: Most evidence comes from controlled research environments with restricted inclusion criteria.
- Imprecision: Heterogeneity in therapist fidelity (i.e. how consistently they apply to treatment protocols) and client adherence leads to wide confidence intervals, lowering certainty in effect estimates. Importantly, applying updated GRADE approach to explicitly assess imprecision helps move beyond purely statistical interpretations of effect estimates. By checking whether confidence intervals cross the threshold for clinical importance, reviewers can better capture practical uncertainty. In meta-analyses of CBT for depression, for instance, inadequate fidelity monitoring often leads to highly variable outcomes, warranting downgrading for imprecision when intervals include both clinically important and trivial effects. This underscores the need to integrate implementation science into psychotherapy research to improve fidelity monitoring and strengthen real-world credibility (21).
- Patients’ values and preferences: There may be several trials prioritizing symptom scales over outcomes that reflect meaningful change in people’s lives, such as quality of life or functional recovery, limiting their relevance to real-world practice and hindering truly patient-centered care.
- Acceptability: There is variable engagement and satisfaction across cultural groups.
- Feasibility: limited access to trained therapists, as reported in implementation studies and observational evidence from low-resource settings.
- Equity: People experiencing health inequities are usually underrepresented in the evidence base (9)

The resulting judgment may classify CBT as “supported with contextual concerns”, indicating strong empirical backing but constrained generalizability. This classification would not diminish the value of CBT but would clarify where and for whom the evidence is strongest and highlight the need for contextual adaptation while implementing it. The process exemplifies how the Integrated Framework transforms evidence evaluation from a static label into a dynamic tool for guiding equitable, culturally responsive implementation (**Box 1**).

Box 1 **Cognitive behavioral therapy as a paradigmatic example**

 Cognitive Behavioral Therapy (CBT) for depression is widely regarded as a prototypical example of an empirically supported psychological treatment. Under traditional EST frameworks, CBT is commonly classified as “well established,” based on a large body of randomized controlled trials demonstrating efficacy in reducing depressive symptoms.

When evaluated using the Contextualized Empirically Supported Treatment Framework (C-EST), this classification becomes more nuanced. Although the certainty of evidence for efficacy remains moderate to high, contextual appraisal highlights limitations related to generalizability, outcome relevance, acceptability, feasibility, and equity across real-world settings.

As a result, CBT may be classified as “supported with contextual concerns,” preserving its strong empirical foundation while explicitly documenting its contextual boundaries. This example illustrates how the C-EST framework refines, rather than weakens, the interpretation of empirically supported treatments by linking certainty of evidence to real-world applicability.

To further operationalize the framework, **Supplementary Table 1** outlines the key contextual domains considered in the C-EST, typical sources of evidence informing each domain, guiding questions for appraisal, and their potential impact on final judgments.

To clarify how the Contextualized EST Framework (C-EST) departs from traditional evaluation models, **Table 1** contrasts key features of conventional Empirically Supported Treatment (EST) approaches with the proposed framework.

**Table 1:** Comparison between traditional empirically supported treatment (EST) evaluation and the integrated EST-contextual framework.

Domain	Traditional EST evaluation	Integrated EST–contextual framework
Primary focus	Efficacy under controlled conditions	Efficacy integrated with real-world relevance
Type of evidence prioritized	Randomized controlled trials	Quantitative and qualitative evidence
Assessment of certainty	Implicit or study-level	Explicit, body-of-evidence assessment using GRADE
Risk of bias and fidelity	Considered, often inconsistently	Systematically appraised using validated tools and transparency standards
Outcomes considered	Primarily symptom reduction	Symptoms plus functioning, well-being, relational and culturally relevant outcomes
Patients’ values and preferences	Not systematically assessed	Explicitly incorporated using EtD and qualitative evidence
Acceptability	Assumed or implicit	Empirically assessed (e.g., engagement, satisfaction, therapeutic relationship)
Feasibility	Rarely considered	Systematically evaluated (resources, delivery format, access barriers)
Equity considerations	Not assessed	Explicit appraisal of representation, access barriers, and impact on disadvantaged populations
Contextual adaptation	Often viewed as protocol deviation	Recognized as legitimate, documentable, and evaluable
Final classification	Binary (e.g., “supported” vs “not supported”)	Nuanced (e.g., “supported with contextual concerns”)
Updating over time	Static classifications	Living evidence model with continuous updating
Decision usefulness	Limited guidance for implementation	High relevance for clinicians, guideline developers, and policymakers

While paradigmatic examples such as Cognitive Behavioral Therapy for depression illustrate how contextual appraisal refines established evidence classifications, some clinical conditions more clearly expose the limitations of efficacy-focused evaluation models. Postpartum depression (PPD) represents a particularly instructive case, as treatment effectiveness is deeply shaped by social context, caregiving demands, health system constraints, and equity-related factors. Values and preferences judgments should be informed, whenever possible, by structured evidence derived from quantitative preference elicitation methods (e.g., health utilities, discrete choice experiments) and qualitative evidence syntheses capturing patient perspectives on outcomes and trade-offs. Examining psychological interventions for PPD through the Contextualized EST Framework (C-EST) highlights how integrating contextual evidence fundamentally alters the interpretation and practical relevance of empirical findings.

## Illustrative example: psychological interventions for postpartum depression

Postpartum depression (PPD) provides a particularly instructive example, drawing on both randomized controlled trials and qualitative evidence syntheses. PPD affects women during a period marked by biological changes, caregiving demands, relational transitions, and heightened vulnerability to social stressors. These features make PPD an archetypal condition in which context fundamentally shapes both need and response to treatment.

## Traditional EST evaluation

Within traditional EST frameworks, psychological interventions, particularly cognitive CBT, for PPD are typically classified as effective. This classification is based on multiple RCTs demonstrating reductions in depressive symptoms compared with usual care or waitlist controls. From an efficacy standpoint, this conclusion is well supported.

However, such evaluations implicitly assume that symptom reduction is the primary outcome of interest, that intervention formats are broadly acceptable, and that implementation barriers are secondary concerns. These assumptions become problematic when translated into real-world postpartum care.

## Contextual evidence from qualitative research

Qualitative systematic reviews of women’s experiences with psychological treatments for PPD provide a richer understanding of how interventions are perceived and used.

Syntheses assessed using GRADE-CERQual consistently identify themes across domains highly relevant to decision-making (22). The qualitative synthesis (22), including studies across multiple countries and care settings, consistently identified themes related to barriers in access, variability in preferences for treatment format, and the centrality of relational and functional outcomes.

Women frequently describe emotional well-being, functional recovery, maternal confidence, and improvements in the mother-infant relationship as central indicators of benefit, often valuing these outcomes more than changes in depression scale scores. This can directly inform judgments about outcome importance.

## Acceptability

Acceptability is strongly shaped by the therapeutic relationship. Women emphasize the importance of empathic, non-judgmental clinicians who understand the postpartum context and avoid pathologizing maternal distress. Preferences regarding treatment format vary widely, with individual, group-based, home-based, and digital interventions each offering advantages depending on personal circumstances (22).

## Feasibility and structural constraints

Feasibility considerations are particularly salient in PPD. Childcare responsibilities, transportation difficulties, fatigue, and time constraints frequently limit women’s ability to engage in traditional clinic-based interventions. Studies consistently report higher perceived feasibility for home-based and digitally delivered treatments, highlighting the importance of flexible delivery models.

## Equity considerations

From an equity perspective, the evidence base for psychological treatments for PPD is disproportionately derived from high-income countries and socially advantaged populations. Women experiencing socioeconomic disadvantage, migration-related barriers, or limited access to services are underrepresented, despite facing elevated risk of PPD and structural barriers to care. This imbalance raises concerns about the generalizability of efficacy findings and underscores the need for equity-sensitive appraisal.

## Integrated judgment

To ensure consistency with the C-EST decision pathway (**Figure 1**), the appraisal follows the same structured steps:

Step 1: Identification of evidence base.

Randomized controlled trials and systematic reviews of psychological interventions for postpartum depression, alongside qualitative evidence syntheses exploring patient experiences (17) were used as the primary evidence base.

Step 2: Certainty of evidence assessment.

Moderate certainty of evidence for reduction in depressive symptoms, based on findings from randomized controlled trials and systematic reviews, supported by qualitative evidence on patient experiences and outcomes considered important by women (21).

Step 3: Assessment of contextual domains.

Values and preferences: Qualitative evidence consistently indicates that women prioritize relational and functional outcomes (e.g., maternal confidence, mother-infant relationship) over symptom reduction alone (22), supporting moderate concerns regarding outcome alignment.

Acceptability: Variability in acceptance of treatment format and the central role of the therapeutic relationship across studies support moderate concerns regarding acceptability (19, 22)
Feasibility: Consistent reports of barriers related to childcare, time constraints, and access to services support moderate to serious feasibility concerns (10, 11, 22)
Equity: The predominance of evidence from high-income and socially advantaged populations supports concerns regarding representativeness and equity (14, 22)

Step 4: Final classification.

Supported with contextual concerns.

This classification was assigned because moderate to serious concerns were identified across contextual domains (acceptability, feasibility, and equity), despite moderate certainty of evidence of benefit, following the predefined C-EST decision criteria.

This stepwise structure ensures consistency with the C-EST framework and makes the classification process transparent and reproducible. This level of detail is intended to illustrate how evidence is translated into judgments within the C-EST framework, rather than to provide a full systematic synthesis. Key implications of the C-EST framework are summarized in **Box 2**.

Box 2 **What changes when psychological treatments are evaluated using the Contextualized Empirically Supported Treatment Framework (C-EST)?**

- Moves beyond binary classifications (“supported” vs. “unsupported”) by allowing nuanced judgments that reflect both certainty of evidence and contextual applicability.
- Requires explicit documentation of contextual boundaries, including limitations related to values and preferences, acceptability, feasibility, cultural relevance, and equity.
- Provides stronger guidance for real-world implementation, supporting clinicians, guideline developers, and policymakers in adapting interventions to diverse settings and populations.
- Aligns empirical evidence with equity and clinical practice, ensuring that judgments about effectiveness are grounded not only in methodological rigor but also in social and ethical relevance.

When assessed using the Integrated EST–Contextual Framework, psychological interventions for PPD would be classified as “supported with contextual concerns.” This judgment preserves the strong empirical evidence for efficacy while explicitly acknowledging that real-world effectiveness depends on alignment with women’s values, feasible delivery formats, and attention to equity gaps.

Under the C-EST framework, maintaining a ‘well-established’ classification without documenting these contextual limitations would represent an incomplete and potentially misleading use of the evidence base.

To illustrate how integrating contextual evidence alters the interpretation of empirical findings, **Supplementary Table 2** contrasts traditional EST evaluation with the Contextualized EST Framework when applied to psychological interventions for postpartum depression. The comparison highlights how consideration of values, acceptability, feasibility, and equity reshapes evidence appraisal beyond efficacy alone.

This logic parallels observations from broader psychotherapy research, such as studies of CBT for depression in diverse community settings, where strong efficacy evidence coexists with variability in engagement and outcomes when contextual factors are not addressed. The PPD example, however, offers a particularly clear demonstration of how context reshapes evidence interpretation.

## Implications for future evidence-based mental health practice

Importantly, the C-EST framework should not be interpreted as an implementation framework. Rather, it operates upstream of implementation, structuring how evidence is appraised and classified before decisions about delivery are made. In this sense, it complements implementation science frameworks, such as PARIHS, which assume that evidence has already been evaluated.

The integration of empirical and contextual perspectives represents a necessary evolution in evidence-based mental health. Integrating contextual appraisal strengthens the relevance of evidence for real-world decision-making.

Three practical implications follow:

1. Guideline development - The Integrated Framework provides a transparent structure for integrating contextual evidence into clinical practice guidelines. Aligning with GRADE and JBI principles, it enables multidisciplinary panels to balance empirical certainty with acceptability, feasibility, and equity in mental health policy.
2. Training and capacity building - Incorporating this concept into the training of psychologists and researchers can promote a new generation of evidence users and producers capable of interpreting both quantitative and contextual data.
3. Implementation and evaluation - The framework encourages living evidence models, fostering continuous updates and shared learning through open repositories. It aligns with current trends in open science and reproducibility.

As the field enters a new phase of evidence-based care, frameworks that provide operational guidance for integrating contextual evidence into evidence appraisal are essential to ensure that evidence not only demonstrates efficacy, but also guides equitable, patient-centered, and implementation-relevant decisions. The Contextualized Empirically Supported Treatment Framework (C-EST) redefines what it means for a psychological treatment to be supported by evidence in real-world mental health care. Future research should apply the C-EST framework in reproducible empirical contexts to further refine and validate its use.

## Implications for clinical practice, guidelines, and policy clinical practice

For clinicians, the framework provides a structured way to integrate empirical evidence with patient values and contextual realities. Rather than treating contextual adaptations as departures from evidence-based care, clinicians can view them as evidence-informed decisions aligned with patient-centered practice.

## Clinical practice guidelines

For guideline developers, the framework aligns psychological treatment evaluation with established health guideline methodology. Explicit consideration of acceptability, feasibility, and equity can reduce the risk of issuing recommendations that are theoretically sound but practically unworkable.

## Policy and service design

For policymakers, the framework highlights that decisions about scaling interventions must consider contextual fit and equity impact. Investing in interventions without addressing delivery constraints may perpetuate disparities rather than reduce them.

## Conclusions and future directions

Evidence-based mental health practice cannot advance by refining methods alone. It must also confront how evidence is interpreted, contextualized, and applied. The C-EST framework offers a concrete pathway for aligning rigor with relevance, ensuring that psychological treatments are not only empirically supported but also responsive to unmet mental health needs across diverse populations. In doing so, it advances evidence-based care that is meaningfully applicable, equitable, and responsive to the realities of patients’ lives.

## Data Availability

The original contributions presented in the study are included in the article/**Supplementary Material**. Further inquiries can be directed to the corresponding authors.
