# Supplementary material for: Beyond empirically supported treatments: a new contextualized evidence framework for evidence based psychology
Source: Front Psychiatry. 2026 May 28;17:1819583. doi: 10.3389/fpsyt.2026.1819583 (PMC13254764; doi:10.3389/fpsyt.2026.1819583)
Supplement: Supplementary file 1 [file Table1.docx]

**Supplementary files**

**Supplementary Table S1.** Operationalization of Contextual Domains in the Contextualized EST Framework (C-EST)

| **Domain** | **Evidence Source** | **Key Questions** | **Judgment criteria** | **Impact on Judgment** |
| --- | --- | --- | --- | --- |
| Values and preferences | Quantitative preference studies (e.g., utilities, discrete choice experiments), qualitative evidence syntheses, mixed-methods studies | How do individuals value outcomes associated with the intervention? What trade-offs are they willing to make between benefits and harms? | - Relative importance assigned to outcomes (e.g., quality of life)  - Willingness to accept trade-offs between benefits and harms  - Variability in preferences across individuals or populations | Preference-sensitive decisions when variability or uncertainty in values and preferences is present |
| Acceptability | Qualitative evidence syntheses, mixed-methods studies, interest-holder perspectives | How do individuals perceive the intervention? What emotional, cultural, or cognitive factors influence engagement? | Emotional responses (e.g., fear, anxiety), perceived burden (e.g., inconvenience), alignment with personal or cultural values, understanding of the intervention, perceived effectiveness, opportunity costs, and self-efficacy | ↓ uptake; need for adaptation or contextualization |
| Feasibility | Observational studies, implementation studies, health system data, qualitative/ mixed-methods evidence | Can the intervention be implemented, accessed, and sustained within real-world settings? | - Individual-level: time constraints, transportation, financial costs, ability to navigate services  - Organizational-level: workforce capacity, service organization, waiting times, infrastructure  - System-level: scalability, geographic coverage, resource availability, sustainability | ↓ implementability; context-specific adaptation required |
| Equity | Representation in studies; subgroup analyses; baseline risk data; observational and implementation studies; qualitative evidence and lived experience; PROGRESS-Plus factors | Who is underrepresented or excluded? Are there differences in effects or access across populations? Could the intervention reduce or exacerbate existing inequities? | Representation of disadvantaged populations; differences in baseline risk or effects; presence of access barriers; contextual factors affecting implementation | ↓ generalizability; conditional use; potential modification of recommendations |

This table provides an operational guide for assessing contextual domains within the C-EST framework. Judgments should be based on the synthesis of available quantitative and qualitative evidence, using signaling questions adapted from GRADE Evidence-to-Decision frameworks. The table is intended to support transparent and reproducible judgments, rather than to function as a rigid checklist or scoring system.

**Supplementary Table S2.** What changes when contextual evidence is integrated? The case of postpartum depression

| **Domain** | **Traditional EST evaluation** | **Integrated EST–Contextual Framework** |
| --- | --- | --- |
| **Efficacy** | **CBT reduces depressive symptoms** | **CBT reduces depressive symptoms** |
| **Values (importance of outcomes)** | Not explicitly evaluated | High importance placed on emotional well-being, functional recovery, and mother-infant relationship |
| **Acceptability** | Implicit or assumed | Strongly influenced by therapeutic relationship, non-judgmental care, and acceptability of treatment type and format |
| **Feasibility** | Not systematically considered | Practical barriers (childcare, time, transport) are central; home-based and digital formats enhance feasibility |
| **Equity** | Not assessed | Evidence largely from high-income settings; limited representation of populations at risk of experiencing inequities |
